# Co-transformation of Aspergillus fumigatus: a simple and efficient strategy for gene editing without linking selectable markers

**DOI:** 10.1099/acmi.0.001057.v3

**Published:** 2025-10-13

**Authors:** Bridget E. Walker, Elaine Bignell, Alex Andrianopoulos

**Affiliations:** 1 School of BioSciences, University of Melbourne, Victoria, Australia; 2 Department of Biosciences, Faculty of Health and Life Sciences, Medical Research Council Centre for Medical Mycology at the University of Exeter, Exeter, UK

**Keywords:** *Aspergillus fumigatus*, DNA-mediated transformation, selectable markers, targeted DNA modifications

## Abstract

Understanding the basis of fungal pathogenesis requires knowledge of pathogen biology that is built through studies of gene function and regulation. The critical first step in nearly all these studies is genetic transformation: the generation of targeted DNA sequence modifications through the introduction of exogenous DNA into the cell. For research focused on gene regulation, or where small precise mutations are desired, the maintenance of genomic context (i.e. surrounding DNA sequences) is important, as the disruption of flanking DNA elements can alter gene expression and confound results. This often makes the inclusion of selectable markers that are physically linked to the sequence of interest unsuitable and complicates the transformation process. Here, we present a co-transformation strategy in the human pathogen *Aspergillus fumigatus* that can be used to make precise, marker-free gene edits at a locus of interest without disturbing flanking DNA sequences. By simultaneously introducing a marker-free, modified copy of the gene of interest and a plasmid that directs the integration of a selectable marker to a different locus, this approach takes advantage of the benefits of selection, with results similar to that of a truly markerless strategy.

## Data Summary

The authors confirm all supporting data, code and protocols have been provided within the article or through supplementary data files.

## Introduction

*Aspergillus fumigatus* is a globally prevalent human fungal pathogen and has been flagged as a critical threat to human health by the World Health Organization [1,2]. The fungus is the causative agent of several serious human infections, including invasive aspergillosis, a life-threatening condition that claims the lives of over 1.8 million individuals annually [3,4]. Studies investigating the basis of *A. fumigatus* pathogenicity are necessary to inform the development of novel therapeutic agents and strategies to prevent and treat infections. Such research relies heavily on available gene-editing tools and methodologies. Improvements to these existing methods and/or the addition of extra tools are therefore highly valuable.

One of the most important methods in genetic research is nucleic acid-mediated transformation, for which selectable markers play a crucial role. There is a clear benefit to incorporating a selectable marker within the DNA construct used for transformation, as it permits easy isolation of cells that have integrated this DNA into their genome through selection on medium supplemented with the selective agent. Dominant genes such as the *Aspergillus oryzae* pyrithiamine resistance gene (*ptrA*) and the *Escherichia coli* hygromycin B phosphotransferase gene (*hph*) have been extensively used for this purpose [5,7]. The downside of incorporating markers is that, in the vast majority of cases, these genes and their regulatory elements are then physically linked to the genetic change one seeks to introduce. This may be an issue when generating precise changes in genes where wild-type expression patterns are ideally maintained and where flanking genomic sequences may influence this expression. Linking a selectable marker to the genetic modification also complicates both the design and the construction of gene replacement cassettes.

*In vitro* assembled CRISPR-Cas9 systems have been shown to be effective for introducing changes into the *A. fumigatus* genome without selection, allowing for wild-type expression to be maintained [8]. This strategy does, however, require Cas9 nucleases, guide RNA components and appropriate buffers, which can become expensive if needed repeatedly. It can also necessitate screening large numbers of isolates to identify clones of interest due to the background of untransformed cells that grow in the absence of selection.

In this article, we highlight an alternative strategy for overcoming the selectable marker-linkage problem, namely co-transformation. The term ‘co-transformation’ refers to the simultaneous introduction of two or more DNA molecules during transformation. In the context of making changes without linking a selectable marker, these DNAs are a modified copy of the gene of interest and a selectable marker. This strategy, which does not require any additional reagents beyond those used in standard fungal transformation protocols, was widely used in the 1980s and 1990s in species such as *Aspergillus nidulans* and *Saccharomyces cerevisiae* [9,10]. It was also recently tested in *A. fumigatus*, although only in conjunction with CRISPR [8]. The advantage of introducing an unlinked selectable marker over a completely marker-free strategy is that the pool of potential transformants can be narrowed down by selection of those that have at least integrated one of the introduced DNA molecules into their genome before screening for integration of the second construct.

We have tested a co-transformation strategy in a non-homologous end joining-impaired (NHEJ-impaired) genetic background, using the pSK379 plasmid, which targets the *ptrA* selectable marker (conferring pyrithiamine resistance) to the 3′ region of the *his2A* gene [11], and a linear DNA fragment containing a modified allele of the *creA* gene, a key regulator of carbon metabolism. Transformants were selected for pyrithiamine resistance and subsequently screened for changes at the *creA* locus. On average, 11% of the selected *ptrA^+^* transformants were found to have been modified at the second locus, an efficiency comparable to CRISPR-based marker-free gene-editing methods [8]. The results indicate that co-transformation is a simple, inexpensive and efficient method for introducing unlinked, ‘marker-free’ changes in the *A. fumigatus* genome that complements the current toolset for genetic modification strategies in this species and may be useful in other species. The strategy also opens the possibility of making multiple desired mutations in one step.

## Methods

### Strains and plasmids

An *A. fumigatus ΔcreA* strain (MFIG TFKO library 1G11) kindly provided by the Manchester Fungal Infection Group [12,13] was used as the recipient strain for transformation. This strain was generated in the *ΔakuB* laboratory strain background, with the only difference being that the *creA* coding sequence is replaced with a hygromycin resistance cassette. Two DNA molecules were introduced in the transformations: pSK379, a *his2A-*targeting plasmid that was transformed in circular form, and the linear *creA^M^* construct, which was amplified from the pBW005 plasmid using primers AfCreA_5′_F and AfCreA_3′_R (Table S1, available in the online Supplementary Material). Both plasmids are described in Table 1. *Talaromyces marneffei* and *A. fumigatus creA* sequences are available in GenBank, and primers used to generate pBW005 are listed in Table S1.

**Table 1.** Plasmids used in this study

Name	Description	Reference/Source
pSK379	Integrative plasmid containing *gpdA* promoter, *A. fumigatus his2A*-targeting sequence and *ptrA* selectable marker in pBluescript II KS(+).	Szewczyk and Krappmann [11]
pBW005	*T. marneffei creA* coding sequence flanked by *A. fumigatus creA* 5′ and 3′ homology arms (−1,006 bp relative to START codon, and +1,041 bp relative to STOP codon) in pBluescript SK(+).	This study

### Transformation and growth conditions

Protoplast-mediated transformation was based on previously described methods for *A. nidulans* [14,15]. Conidia (2×10^8^) of the *ΔcreA* strain were inoculated into 40 ml of Sabouraud Dextrose liquid medium (SAB; 2% w/v Dextrose, 0.5% w/v Pancreatic digest of casein, 0.5% w/v Peptic digest of animal tissue, Oxoid^™^) in a 250 ml Erlenmeyer flask and incubated with shaking (130 r.p.m.) for 16 h at 30 °C. The hyphal culture was harvested through sterile Miracloth and resuspended in a new 250 ml flask with 16 ml of fresh SAB and 16 ml of filter-sterilized (0.22 µm PVDF-filter) 2× protoplasting solution [2.05 g VinoTaste Pro in 16 ml of KCl citric acid solution (1.1 M KCl and 0.1 M citric acid in Milli-Q water adjusted to pH 5.8 with 1.1 M KOH)]. This flask was incubated with shaking (100 r.p.m.) for 2 h at 30 °C for cell wall digestion. The protoplasting culture was then filtered through a sterile 40 µm cell strainer (Fisherbrand^™^) to remove undigested hyphal material and the filtrate pelleted by centrifugation for 10 min at 1,800 ***g*** at 4 °C. The protoplast pellet was washed thrice with 1 ml of 0.6 M KCl and centrifuged for 3 min at 2,400 ***g*** at 4 °C before being finally resuspended in 0.6 M KCl and 50 mM CaCl_2_. Protoplast concentration was determined using a haemocytometer, and the protoplast suspension was diluted to 5×10^6^ protoplasts ml^−1^. For the transformation reaction, 2 µg of both pSK379 and the *creA^M^* construct (in no more than 25 µl TE buffer), 200 µl of the diluted protoplast suspension and 100 µl of filter-sterilized (0.22 µm PVDF-filter) PEG solution (25% w/v PEG, 0.6 M KCl, 50 mM CaCl2 and 1M Tris/HCl pH 7.5) were added to a sterile microfuge tube, mixed and incubated on ice for 30 min. 1.25 ml of PEG solution was then added and mixed by pipetting and the reaction mixture was incubated at room temperature for 45 min – 1 h. The reaction mixture (total volume ~1.6 ml) was then mixed with molten (cool to touch) regeneration medium (RM) supplemented with 500 µg ml^−1^ pyrithiamine and poured across eight plates (~200 µl reaction mix plated per plate). RM medium is Aspergillus minimal medium (AMM) [16] containing 50 mM ammonium tartrate as a nitrogen source and 1 M sucrose as an osmotic stabilizer. Plates were incubated overnight at room temperature and transferred to 37 °C the following morning. After 3 days, transformant colonies were visible on the selection plates. Colonies were screened for loss of the *hph* marker on Sabouraud Dextrose agar supplemented with 200 µg ml^−1^ hygromycin B. Hygromycin-sensitive transformants were purified by streaking for single colonies on AMM supplemented with 500 µg ml^−1^ pyrithiamine. Genomic DNA (gDNA) was extracted from four transformants: frozen mycelia were lysed in lysis buffer (50 mM Tris/HCl pH 8, 50 mM EDTA pH 8, 3% w/v SDS and 1% v/v 2-mercaptoethanol) using a FastPrep-24^™^ 5G bead beating grinder and lysis system (MP Biomedicals). DNA was then purified using 25:24:1 Phenol-Chloroform-Isoamyl Alcohol [17]. Successful integration of the *creA^M^* construct was then validated by PCR and XbaI restriction enzyme digestion of the PCR products. PCR mixes (25 µl) comprised 1× *Taq* buffer, 100 µM dNTP mix, ~30 ng of gDNA, 0.2 µM of each primer (AfCreA_5′_F and AfCreA_3′_R) and 0.625 units of *Taq* DNA polymerase. Thermocycling conditions were 95 °C for 20 s, 55 °C for 30 s and 68 °C for 5 min for 30 cycles. PCR products were purified using the Bioneer Accuprep^®^ PCR purification kit, and ~150 ng of each purified PCR product was digested with XbaI restriction enzyme (0.5 units μl^−1^) for 4 h at 37 °C.

## Results

The potential utility of co-transformation was assessed while studying CreA, a key regulator of carbon metabolism, which has previously been linked to *A. fumigatus* virulence [13,18]. We were interested in the function of CreA orthologues in filamentous fungi and whether amino acid sequence conservation was indicative of functional conservation (i.e. whether or not the CreA protein from one species could substitute for another). We aimed to generate a strain of *A. fumigatus* in which the *AfcreA* coding region was replaced with the *creA* coding region of *T. marneffei*, a related fungal pathogen, and examine whether this strain could phenocopy the *A. fumigatus* wild-type strain with respect to virulence and carbon source utilization. The modified *A. fumigatus creA* construct (*creA^M^*) comprised the *TmcreA* coding sequence flanked by the *A. fumigatus* 5′UTR and 3′UTR. With the focus of this project being solely on protein function, it was necessary to ensure that the results of this study would not be influenced by changes in gene expression or mRNA stability that may occur through the linkage of a selectable marker. To generate this allele, a *ΔcreA* strain was used as the recipient strain. This strain contained an *hph* selectable marker in place of the *creA* coding region at the *creA* locus, thus restricting homologous recombination events to the 5′ and 3′ homology arms of the introduced *creA^M^* construct. The *ΔcreA* strain is a derivative of the A1160^+^ strain (also known as MFIG001), which lacks the *akuB* gene to improve efficiency of homologous recombination events [19,20].

Co-transformation is dependent on a sufficiently large number of protoplasts being competent to take up the DNA constructs. The transformation procedure was first optimized to ensure that co-transformant recovery was not limited by low transformation efficiency. Optimization of the procedure was performed with the A1160^+^ strain by varying cell wall digestion times and protoplast concentrations when introducing only the pSK379 plasmid (containing the *ptrA* selectable marker). Protoplast concentration during transformation and selection had the greatest impact on transformation efficiency, with the greatest number of *ptrA^+^* transformants being recovered when protoplasts were diluted to 5×10^6^ protoplasts ml^−1^ per transformation reaction (Fig. S1). After optimizing the transformation conditions and using this protoplast concentration, an average of 43 *ptrA^+^* transformants were recovered per reaction (21.5 transformants per microgram of DNA per 5×10^5^ protoplasts).

**Fig. 1.**
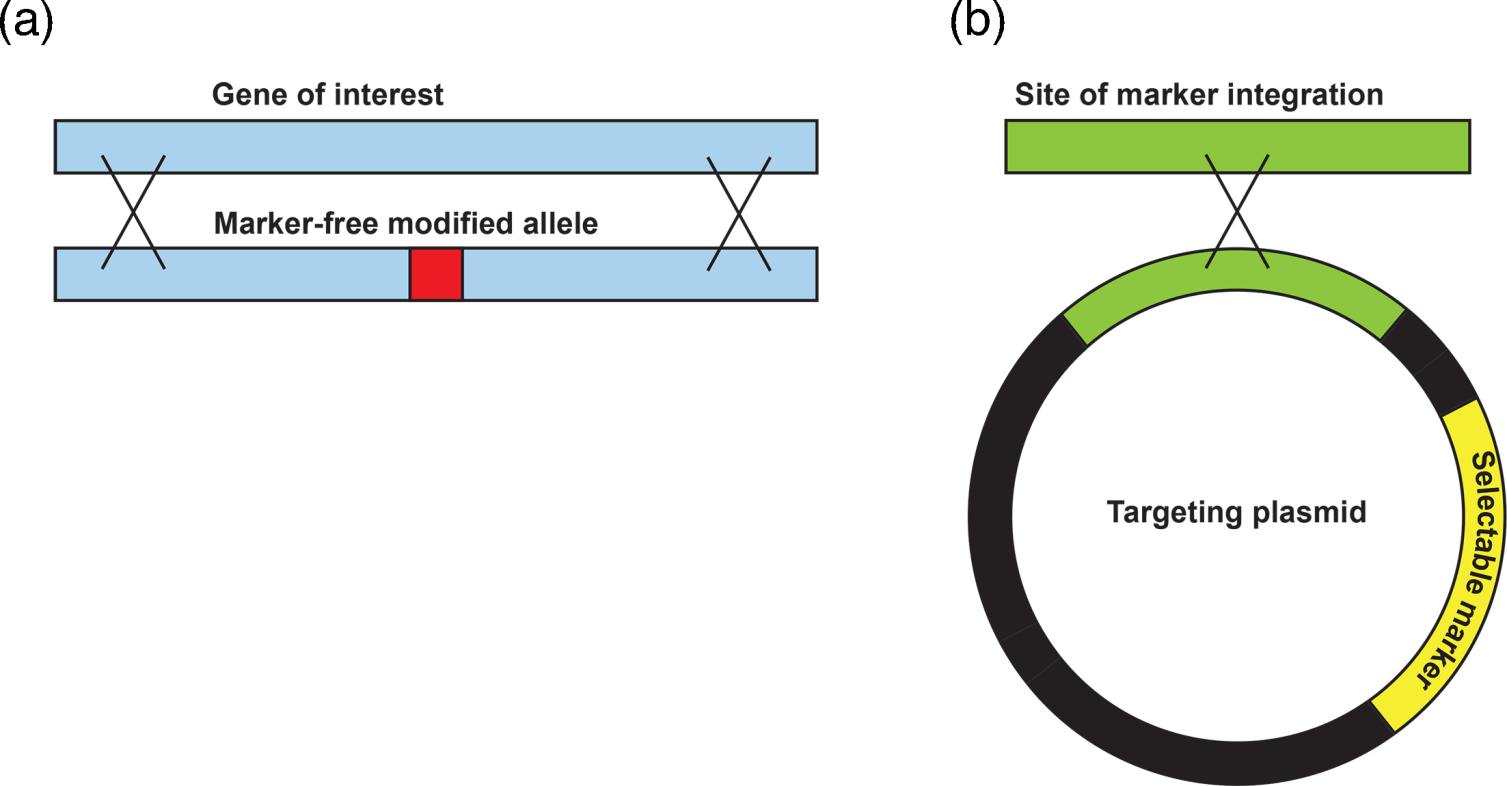
Generalized co-transformation strategy. Illustration depicting simultaneous integration of two DNA molecules into the *A. fumigatus* genome. (**a**) Integration of linear DNA fragment with marker-free genetic modification (red) at the locus of interest (blue) by two homologous recombination events. (**b**) Integration of targeting plasmid containing selectable marker (yellow) into alternate locus (green) by single homologous recombination event.

The co-transformation strategy (Fig. 1) was tested using the same conditions. Co-transformation reactions were performed using a 5:2 molar ratio of target construct (linear *creA^M^*) to selectable marker (*ptrA*, pSK397). The transformation reaction was plated on medium supplemented with 500 µg ml^−1^ pyrithiamine and 139 *ptrA^+^* transformants were recovered from three independent transformation reactions (Table 2). These transformants were subsequently screened on medium supplemented with 200 µg ml^−1^ hygromycin B and 16/139 transformants were identified as exhibiting sensitivity compared to the starting strain, suggesting that they had lost the *hph* selectable marker at the *creA* locus, in addition to having incorporated the *ptrA* marker at the *his2A* locus. gDNA was extracted from four of the pyrithiamine-resistant, hygromycin-sensitive transformants and subjected to diagnostic PCR and restriction digest analysis. Replacement of the *hph* marker with the *creA^M^* construct was confirmed by both PCR and restriction digest in each of these transformants (Fig. S2, Table S2).

**Table 2.** Transformation efficiencies

Reaction	Pyrithiamine-resistant	Pyrithiamine-resistant, hygromycin-sensitive	Co-transformation percentage
1	42	4	9.5
2	50	7	14
3	47	5	10.6

## Discussion

The ability to make precise genetic mutations is a fundamental tool for biological studies of molecular processes. The gene-editing strategy is often dependent on the organism being studied and the type of mutation that is required. Often, it is desirable to make precise mutations at a locus without adding any unnecessary or extraneous DNA to the target locus, for example, in order to maintain genomic context. A number of strategies have been developed for this purpose, including selection-free screening and two-step processes using selectable markers with both positive and negative selection attributes [8,21,23]. The work presented here offers an alternative approach using co-transformation. The method is straightforward, and if the recipient strain is an NHEJ-deficient laboratory strain, no additional genetic alterations are required prior to the transformation.

**Fig. 2.**
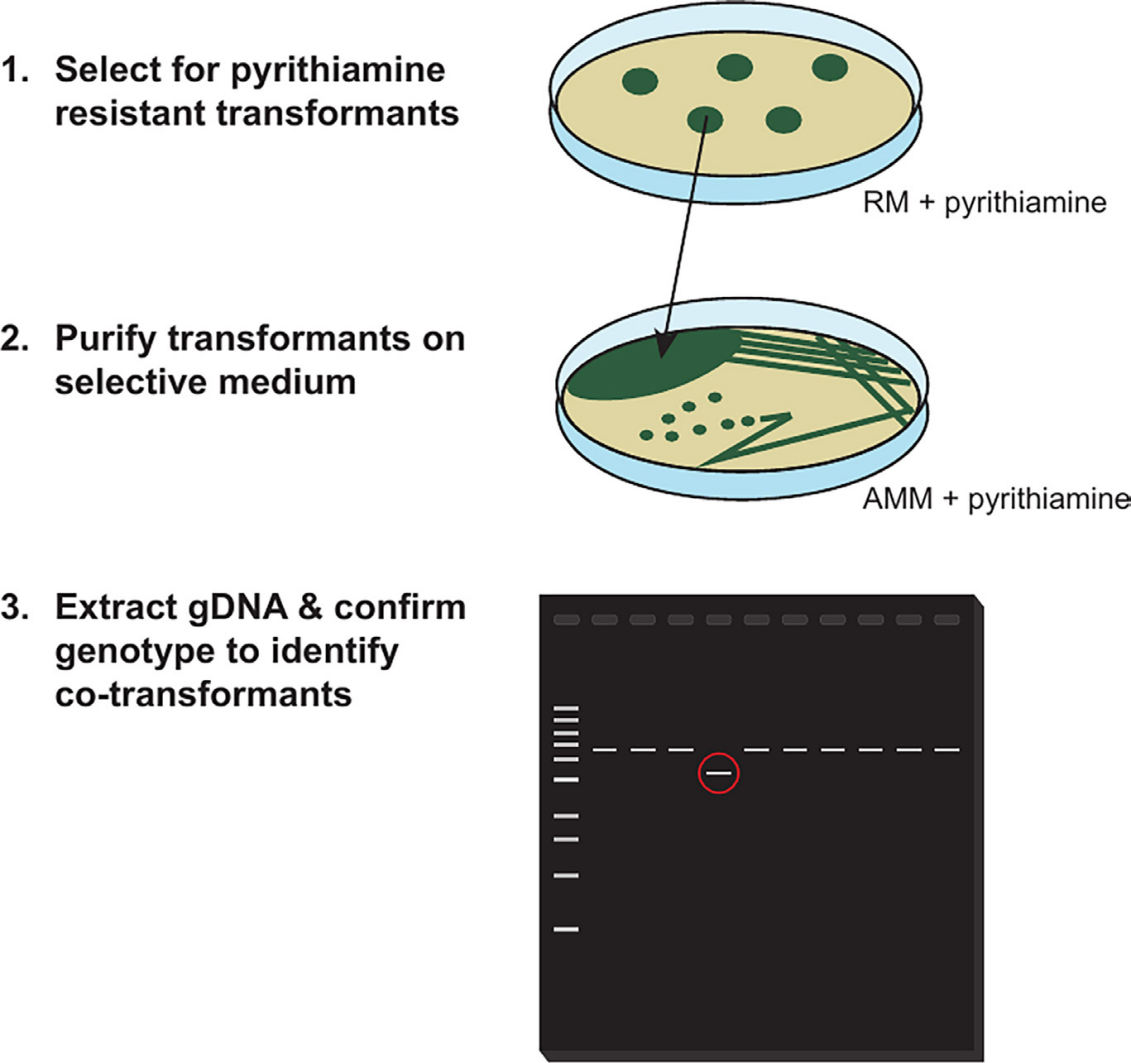
Identification of co-transformants. Pyrithiamine-resistant transformants are selected on RM supplemented with pyrithiamine and then purified by streaking for single colonies on AMM supplemented with pyrithiamine. gDNA is then extracted from purified pyrithiamine-resistant transformants and those that have integrated the marker-free construct at the locus of interest are identified by PCR or other genotyping methods. A red circle indicates the expected product size for a co-transformant; on average, 11% of transformants are successfully co-transformed.

The co-transformation process was tested using an *in vitro* generated allele of the gene of interest (*creA^M^*) and a selectable marker (*ptrA*) targeted to a commonly used locus (*his2A*). Briefly, ~11% of selected pyrithiamine-resistant transformants were co-transformed when using a 2:5 ratio of *his2A*-targeting plasmid and linear *creA^M^* construct. With this frequency, screening of 10–20 selected transformants is generally sufficient for identifying transformants with the genotype of interest. Given that 42–50 *ptrA^+^* transformants were recovered per reaction, screening for transformants with the desired genetic make-up was highly feasible and transformants that had successfully integrated the markerless *creA^M^* construct were readily identified. Good-quality gDNA can be easily extracted from *A. fumigatus* conidia [20], which makes it possible to rapidly screen the selected colonies for the intended gene edit (Fig. 2). These results indicate that co-transformation represents a simple method for generating genetic modifications without needing to link selectable markers to the gene of interest. This strategy has the added advantage of being inexpensive and does not require any additional reagents beyond those needed for a traditional ‘single’ transformation.

It is important to note that the success of this strategy relies on high co-transformation frequencies, which are a product of both the uptake of DNA by the protoplasts and the integration of this exogenous DNA into the genome by homology-directed repair. While this strategy was only tested targeting the *creA* locus, it is highly likely to work for most other loci, noting that frequencies of homologous recombination may differ at other genomic locations, as has been reported in other fungi [24]. In cases where the strategy is used to make small mutations (such as a small deletion or base substitution), the length and positioning of the homology arms in the transforming DNA should also be carefully considered to reduce the likelihood of recombination events not flanking the desired mutational site.

The efficiency of any transformation strategy is affected by the genetic background of the recipient strain. Most fungi, including *A. fumigatus*, use the NHEJ pathway instead of homologous recombination for the repair of dsDNA damage or the integration of exogenous linear DNA fragments [19,25, 26]. Disruption of essential elements of the NHEJ DNA repair pathway helps to favour homologous recombination and dramatically improves the rate at which targeted genetic mutations can be made [19,26,32]. For this reason, NHEJ-impaired laboratory strains are routinely used for genetic research. The *ΔcreA* strain used in this study was NHEJ-impaired due to deletion of the *akuB* gene (also known as *ku80*), which increased the likelihood of the two introduced DNA molecules being integrated into the genome by homologous recombination. We therefore consider the recombination frequencies observed to be representative of *A. fumigatus ΔakuB* strains, specifically A1160^+^, and would expect similar frequencies when co-transforming other NHEJ-impaired genetic backgrounds such as *ΔakuA* [28,33]. Use of the co-transformation strategy in strains with a functional NHEJ pathway is also an option and may be appropriate in some circumstances; however, there is a risk of disruption of other *A. fumigatus* genes, which reduces certainty that observed phenotypes are a result of the intended genetic modification. If having a functional NHEJ pathway is important for the study, it may be preferable to generate the desired mutations in a *ΔakuB* strain by co-transformation with three DNA molecules: the modified gene of interest, the selectable marker and a functional copy of the *akuB* gene to repair the locus and restore NHEJ.

Use of a targeting plasmid for the selectable marker was another key component of the transformation strategy tested. The plasmid used in this study, pSK379, contains both a pyrithiamine resistance marker and a *his2A*-targeting sequence to direct integration of the selectable marker to a 1,997 bp region of the genome, 3′ of the *his2A* gene (Fig. S3). Many studies have used pSK379 or other *his2A*-targeting plasmids, and insertion of exogenous DNA at the *his2A* locus has not been reported to impact fungal physiology or virulence [11,34,38], although it was not specifically examined in any of these studies. Whether insertion of a *ptrA* marker at this site has any downstream impacts cannot be known for sure, making it very important to carefully assess the impact of the *his2A::ptrA* genotype on the phenotypes being studied. Plasmids which target different genomic locations and/or contain different selectable markers (e.g. *hph*) are also likely to work in place of pSK379.

Only one other study appears to have reported a co-transformation strategy in *A. fumigatus* [8]. In this study, co-transformation of an unlinked selectable marker (*hph*) was used in conjunction with an *in vitro* assembled CRISPR-Cas9 genetic replacement system to make ‘marker-free’ changes in an NHEJ-impaired genetic background. The number of CRISPR-edited transformants recovered did not improve when co-transforming and selecting for an unlinked selectable marker (*hph*), compared to those recovered from the ‘single’ CRISPR-Cas9 transformations without selection (~8.5% with co-transformation of a marker versus 10–20% without). A ‘single’ CRISPR-Cas9 transformation is already similar to co-transformation, in that it relies on both the CRISPR machinery and the repair template being taken up by the same protoplast. For the ‘co-transformation’ strategy tested in that study to work, protoplasts had to take up four molecules: two CRISPR-Cas9-gRNA ribonucleoprotein complexes, the repair template and the selectable marker.

Currently, the advantages of using drug-resistant selectable markers still make them very appealing for genetic manipulation. Such selectable genes are usually driven by strong promoters and constitutively expressed. Therefore, the transformed strains are always resistant to the selective agent. If constitutive expression of these markers were to be a concern, it is also possible to modify both *ptrA* and *hph* selection cassettes so they are inducible [39].

This study has demonstrated that co-transformation is an efficient method for the simultaneous integration of multiple DNA fragments into the *A. fumigatus* genome. Co-transformation takes advantage of the benefit of using selectable markers to identify transformants, without the risk of altering gene expression at the target locus due to effects of the linked selectable marker. This strategy has great utility for studies concerned with gene regulation (e.g. the introduction of epitope-tags and manipulation of non-coding regulatory elements) or where wild-type gene expression is ideally maintained (e.g. site-directed mutagenesis).

## Supplementary material

10.1099/acmi.0.001057.v3 Uncited Supplementary Material 1.

## Abbreviations

AMM: Aspergillus minimal medium
gDNA: genomic DNA
NHEJ: non-homologous end joining
RM: regeneration medium
