# Supplementary material for: Co-transformation of Aspergillus fumigatus: a simple and efficient strategy for gene editing without linking selectable markers
Source: Access Microbiol. 2025 Oct 13;7(10):001057.v3. doi: 10.1099/acmi.0.001057.v3 (PMC12517355; doi:10.1099/acmi.0.001057.v3)
Supplement: Uncited Supplementary Material 1. [file acmi-7-01057-s001.pdf]

# Supplementary material

**Table S1. Oligonucleotides used in this study.**

| Primer | Sequence | Use |
|---|---|---|
| AfCreA_5'_F | ATATCGAATTCCTGCAGCCCTGCGTGGTCGTACCAAAATG | Amplification of the *Af creA* 5’ homology arm |
| AfCreA_5'_R | CCACTGACGAAGCTGACGGTGGCATGTGACGCTTGACTTA | |
| TmCreA_CDS_F | GCGTTGAACTAAGTCAAGCGTCACATGCCACCGTCAGC | Amplification of the *Tm creA* coding sequence |
| TmCreA_CDS_R | ATCATGTAGACTCTACCTGATTATAACTCCATCACGGATCCG | |
| AfCreA_3'_Tm_F | GATCCGTGATGGAGTTATAATCAGGTAGAGTCTACATGATATC | Amplification of the *Af creA* 3’ homology arm |
| AfCreA_3'_R | CTAGAACTAGTGGATCCCCCGAAAGCCCGATCAATCCAAG | |

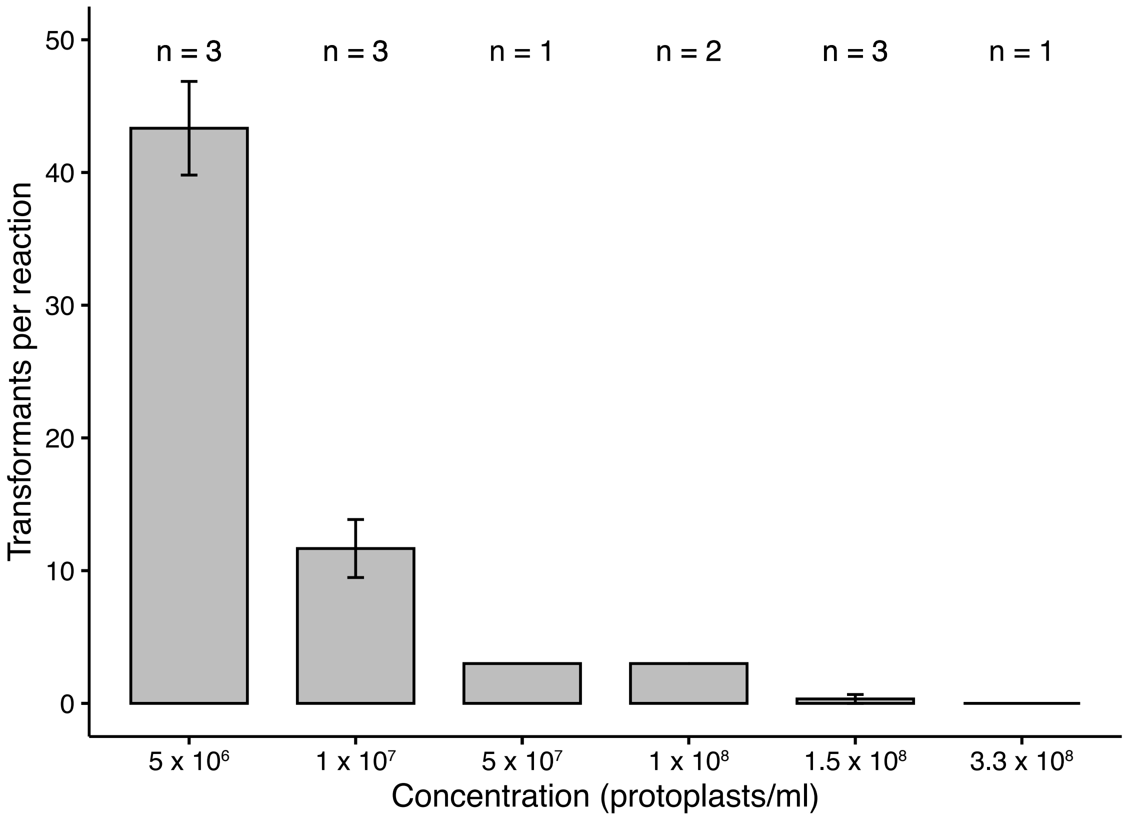


**Figure S1. Impact of protoplast concentration on *Aspergillus fumigatus* transformation efficiency.** Graph depicts number of transformants recovered when transforming different concentration of A1160$^+$ protoplasts with 2 μg of plasmid DNA (pSK379) and selecting for pyrithiamine-resistant transformants. All other reaction parameters, including the volume of protoplast mixture, were kept consistent. The number of replicates (n) are indicated above each bar, and SEM error bars are shown for concentrations where n = 3. The purpose of this experiment was to examine the impact of protoplast concentration on transformation efficiency and determine an optimal concentration. Additional replicates were therefore deemed unnecessary for concentrations were ≤ 3 transformants were recovered.

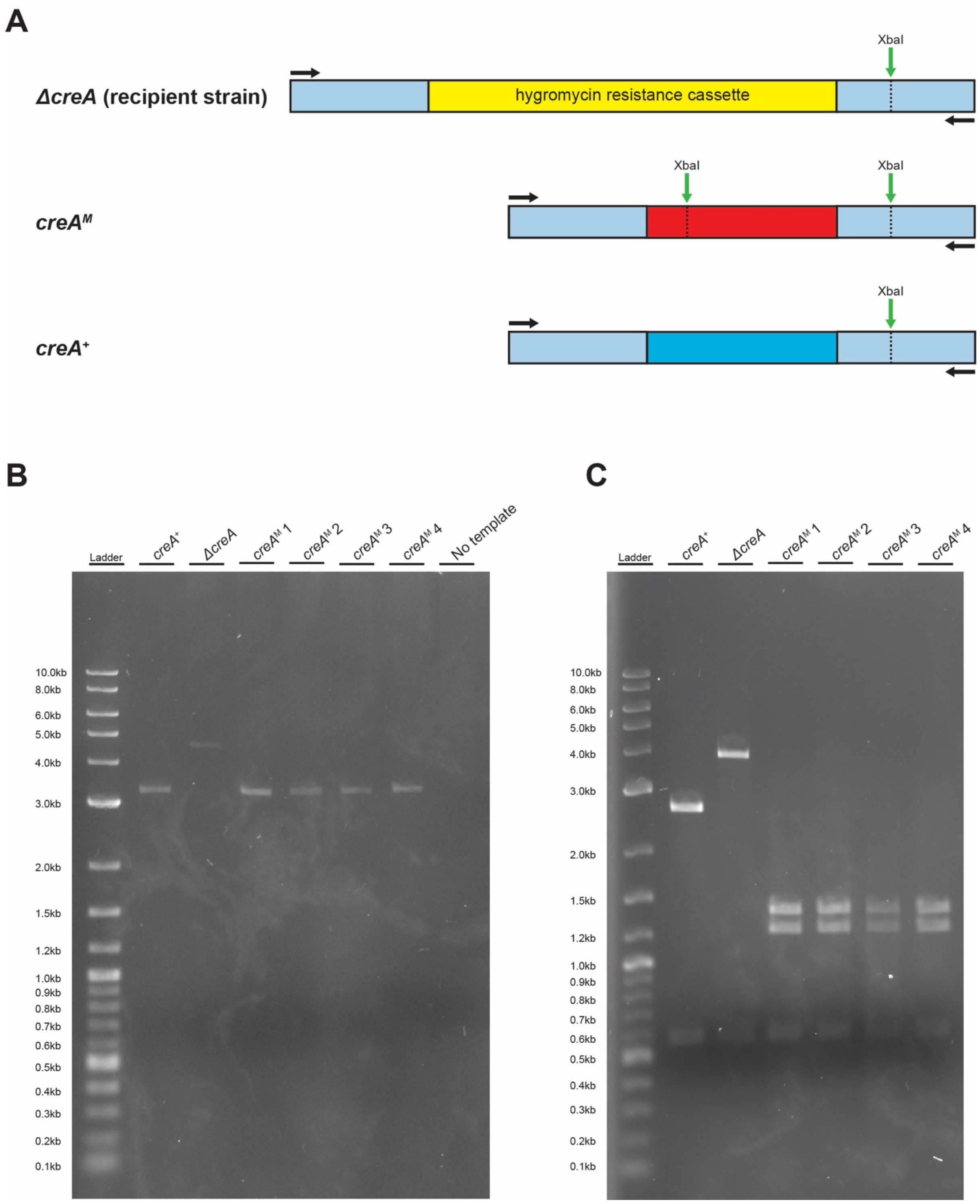


**Figure S2. Confirmation of co-transformants by PCR and restriction digest. (A)** Illustration of genotyping procedure: PCR amplification of the *creA* locus using AfCreA_5'_F and AfCreA_3'_R primers (black arrows) and XbaI restriction enzyme digestion of the PCR products (XbaI restriction sites shown as dashed line and indicated with green arrows). As the *ΔcreA* strain was the recipient strain for the co-transformation, PCR product size alone indicates whether or not the hygromycin resistance cassette had been replaced with the *creA$^M$* construct. The XbaI restriction enzyme digestion was an additional check used to differentiate between the native *A. fumigatus*

*creA* coding region (*creA*$^{+}$) and the modified *creA*$^{M}$ allele due to presence of an additional XbaI restriction site in the *T. marneffei creA* coding region. **(B)** Ethidium bromide-stained 1% agarose gel with DNA fragments produced by PCR amplification of *A. fumigatus creA* genomic DNA and **(C)** Ethidium bromide-stained 1.2% agarose gel with XbaI restriction enzyme digestion of PCR products from (B) to examine the genotypes of putative *his2A::ptrA creA*$^{M}$ co-transformants. Strain/transformant identities are indicated above each lane and Lane 1 of each gel contains the Quick-Load® Purple 1 kb Plus DNA Ladder (NEB). The *creA*$^{+}$ control DNA was derived from the A1160$^{+}$ strain. DNA fragment sizes (listed in table S2) were as expected for all PCR products.

| **Table S2. Expected PCR and restriction digest product sizes.** | | |
|---|---|---|
| **Strain** | **PCR product (bp)** | **XbaI digestion products (bp)** |
| *creA*$^{+}$ | 3362 | 2754, 608 |
| *ΔcreA* | 4792 | 4085, 608 |
| *creA*$^{M}$ | 3330 | 1443, 1279, 608 |

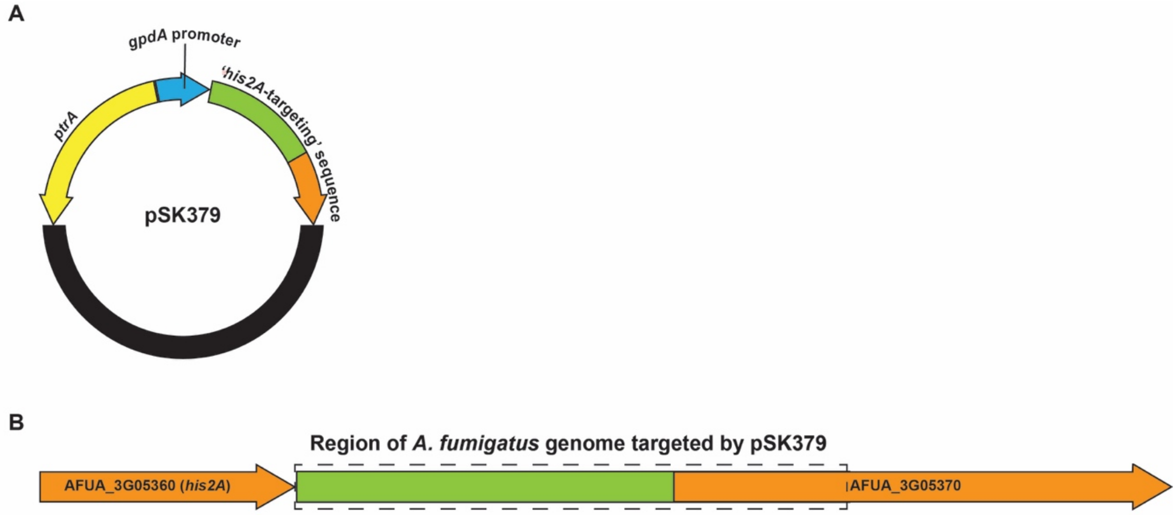


**Figure S3. Map of the *his2A*-targeting plasmid, pSK379, and its site of integration in the *A. fumigatus* genome. (A)** pSK379 contains the *A. oryzae ptrA* selectable marker sequence inclusive of protein-coding and regulatory sequences (2010 bp, yellow), the *A. nidulans gpdA* promoter (435 bp, blue) and a region of homology to the *A. fumigatus* genome, referred to as the '*his2A*-targeting' sequence (1997 bp, green and orange). Plasmid backbone (black) is pBluescript II KS (+). The *gpdA* promoter can be used to drive expression of genes cloned downstream of it. **(B)** Map of the *A. fumigatus* genome region targeted by the pSK379 plasmid with protein-coding sequences shown in orange and non-coding sequence shown in green. The 1997 bp region targeted by pSK379 is indicated by a dashed rectangle, this includes 1317 bp of non-coding DNA 3' of the *his2A* protein-coding sequence, and the first 680 bp of another gene, AFUA_3G05370. Gene/promoter orientations are indicated with arrowheads. Relative sizes of the labelled features are approximations only.
